# Metabolic Regulation of Thymic Epithelial Cell Function

**DOI:** 10.3389/fimmu.2021.636072

**Published:** 2021-03-03

**Authors:** Manpreet K. Semwal, Nicholas E. Jones, Ann V. Griffith

**Affiliations:** Department of Microbiology, Immunology and Molecular Genetics, University of Texas Joe R. and Teresa Lozano Long School of Medicine, UT Health San Antonio, San Antonio, TX, United States

**Keywords:** thymus, thymic stromal cells, mTOR, tolerance, autophagy

## Abstract

The thymus is the primary site of T lymphocyte development, where mutually inductive signaling between lymphoid progenitors and thymic stromal cells directs the progenitors along a well-characterized program of differentiation. Although thymic stromal cells, including thymic epithelial cells (TECs) are critical for the development of T cell-mediated immunity, many aspects of their basic biology have been difficult to resolve because they represent a small fraction of thymus cellularity, and because their isolation requires enzymatic digestion that induces broad physiological changes. These obstacles are especially relevant to the study of metabolic regulation of cell function, since isolation procedures necessarily disrupt metabolic homeostasis. In contrast to the well-characterized relationships between metabolism and intracellular signaling in T cell function during an immune response, metabolic regulation of thymic stromal cell function represents an emerging area of study. Here, we review recent advances in three distinct, but interconnected areas: regulation of mTOR signaling, reactive oxygen species (ROS), and autophagy, with respect to their roles in the establishment and maintenance of the thymic stromal microenvironment.

## Introduction

Appropriate tissue function requires integration of intra- and extracellular signals that govern cellular division, migration, and growth, as well as the regulation of organelle size, macromolecule synthesis, and gene expression. Efficiently carrying out such functions requires a balance of catabolic activity required for energy generation, and anabolic activity required for biogenesis. The mechanisms by which signal transduction pathways downstream of growth factor signaling regulate metabolism to influence cellular energy and redox status are well-characterized (1), and it is now clear that the metabolic pathways employed in a given cell feedback to signal transduction pathways. For instance, metabolite-mediated and ROS-mediated modification of proteins involved in signal transduction may alter their activity, and *de novo* intracellular signaling transduction can be initiated by mitochondrial ROS production (2). These types of metabolic signaling influence fundamental cellular decisions such as quiescence vs. activity (3–5), and stem cell self-renewal vs. differentiation (6–8).

The role of metabolic pathways as regulators of cellular function has become an area of increasing interest over the last decades, and T cells have been a major area of focus (3, 9). Metabolic control of processes such as activation downstream of TCR engagement, and effector functions such as IFNg production have been shown to be mediated by mitochondrial ROS and the glycolytic enzyme GAPDH, respectively, in T cells [reviewed in (3)]. In contrast, relatively little is known about metabolic regulation of thymic stromal cell function. Understanding metabolic regulation of stromal cells is important as a basic feature of their biology, but is particularly relevant for thymic stromal cells for several reasons. First, metabolic function and dysfunction are closely linked to aging (6, 10), and the stromal cells of the thymus are the primary targets of what could be considered among the first hallmarks of aging, thymic atrophy [reviewed in (11)]. Second, integration of metabolic information is critical in controlling cell size and morphology (7, 12), which are uniquely and dynamically regulated in thymic stromal cells (13), directly governing the niches available for T cell generation (14). Moreover, autophagy, in addition to its ubiquitous roles in energy homeostasis and repair of oxidative damage to organelle, also plays an additional role in thymic stromal cells by generating peptide antigens for presentation critical for T cell selection and tolerance induction (15). In this review, we will consider the integration of three aspects of metabolic regulation: mTOR signaling, the redox status of the cell, and autophagy, in the steady-state function and age-associated dysfunction of thymic stromal cells.

## mTOR Signaling in TEC

The mechanistic target of rapamycin, mTOR, plays an integral role in cell growth and proliferation in response to a wide array of environmental cues. mTOR is a serine/threonine protein kinase belonging to the PI3K-related protein Kinases (PIKK) family (16, 17) and is the main catalytic subunit in two distinct complexes named mTOR complex 1 (mTORC1) and mTOR complex 2 (mTORC2). These complexes integrate environmental cues and result in both distinct and common cellular outcomes, with significant crosstalk between mTORC1 and mTORC2 signaling pathways. mTORC1 responds to inputs such as energy status, nutrients, growth factors, oxygen and stress, and promotes biosynthetic pathways. It also inhibits autophagy and other catabolic process. mTORC2 is thought to be activated primarily by growth factor signaling, and, like mTORC1, also promotes anabolic metabolism, proliferation, and survival. In addition, mTORC2 signaling regulates cytoskeletal reorganization, with impacts on cell motility (17).

mTOR signaling appears to be a critical regulator of thymus function, as demonstrated by the pronounced thymic atrophy caused by high dose rapamycin administration (18–20). Rapamycin-induced atrophy has been associated with arrested thymocyte proliferation (18), consistent with its well-characterized immunosuppressive properties (21). However, recent studies have revealed that mTOR signaling is also a critical regulator of thymic stromal cell function.

Liang and colleagues explored the role of mTOR signaling in thymic epithelial cells (TECs) using a tissue-specific knockout mouse model (19). In this system, the mTOR protein is ablated in TEC, resulting in disruption of both mTORC1 and mTORC2 signaling. This genetic ablation resulted in decreases in the number of medullary TECs (mTECs) and cortical TECs (cTECs) during fetal development and at 2 weeks after birth, as well as a reduction in the frequency and number of MTS24^+^ progenitors. Knockout mice also showed decreased proliferation and increased autophagy in TEC, as well as dysregulated T cell development (19). These effects may be explained by disruptions of either mTORC1 or mTORC2.

A pair of recent studies addressed the role of each mTOR complex independently. In mice in which mTORC1 was selectively inhibited in TEC, Wang et al. found that total thymus cellularity, cTEC, and mTEC number decreased. TEC in knockout mice also showed decreased proliferation, and glucose uptake, but TEC survival was not affected. These results are consistent with a role for mTORC1 signaling in TEC proliferation and early growth of the thymus. The effect on cell number was most substantial in mTEC, such that the cTEC frequency in knockout mice was significantly higher than in wildtype mice. The frequency of MHCII high mature cTEC and mTEC were decreased in knockout mice up to ~3 weeks of age, after which the frequency was the same as in wildtype mice, consistent with a role for mTORC1 signaling in the establishment and maturation of the TEC compartment in growth phases (22).

In mice in which mTORC2 was selectively inhibited, total thymus cellularity and TEC cell number were likewise decreased (23). In contrast to mTORC1 deficiency, cTEC and mTEC ratios were not altered in mTORC2 deficient mice, because the average number of both cTEC and mTEC declined (although the cTEC declines were not statistically significant), indicating a potential additional role for mTORC2 signaling in cTEC as well as mTEC. Consistent with this, maturation of cTEC, as indicated by high MHCII, expression was diminished. Although cTEC appeared to be less affected in the knockout mice relative to mTEC, T cell numbers were decreased beginning at the earliest (cortical) stages, consistent with decreased cTEC function (23).

A role for mTOR signaling in cTEC is also supported by our recent confocal imaging study, in which we found a unique cTEC morphology. cTEC morphology is characterized by projections that comprise extensive labyrinths creating compartments within each cTEC that contained up to approximately 100–150 lymphoid cells per cTEC. The overall shape of cTECs was generally similar to a compressed ovoid and they were aligned radially with respect to the capsule (13). In aged mice, cTEC processes collapsed, and this loss of cell size occurred in the absence of changes in cell number, resulting in increased cTEC density. During thymus regeneration cTECs partially recovered their processes and labyrinth morphology, but did not proliferate extensively (13). Thus, the size and shape of cTEC are critical for maintaining overall thymus cellularity with age, as well as the regeneration induced by castration. In order to understand the mechanisms regulating cTEC morphology and size, we mined our transcriptional database to find Reactome pathways (reactome.org) associated with cell signaling that were significantly enriched in cortical stromal cells. We found that 3 of the top 4 most significantly enriched pathways were related to mTOR signaling (13), which, as described above, is well-recognized as a regulator of cell and tissue size via effects on metabolism and cytoskeletal organization (16, 24). When we looked in more detail at changes in mTOR pathway enrichment in cortical stromal cells during aging and regeneration, we found that mTOR signaling pathway enrichment declined with age and increased dramatically in the early stages of regeneration, before falling again as regeneration wanes (13). The expression patterns of key mTOR pathway components likewise support the notion that mTOR signaling in cortical stromal declines with age, and is activated during regeneration. Notably, we find upregulation of the mTORC1 regulator Tsc1, consistent with preferential signaling through the mTORC2 pathway important for cytoskeletal remodeling [reviewed in (12)].

We also investigated potential sources of soluble ligand capable of activating the mTOR pathway. These were either absent or not changed during aging and regeneration in cortical stromal cells, making autocrine signaling unlikely (13). Such soluble ligands could be endocrine-derived, however, the cortex of the thymus (but not the medulla) is immune-privileged and separated by a relatively impermeable blood-thymus barrier (25). These observations indicate that intrathymic paracrine signaling may account for the mTOR activation seen in young mice and during regeneration. We found that several ligands, most notably known TEC regulators IGF1 (26) and FGF21 (27), were both diminished with age, and dynamically upregulated in medullary stromal cells during regeneration, presumably as a response to systemic signals induced by castration.

Together, the literature indicates a critical role for mTOR signaling in regulating TEC development, proliferation, size, and function. mTORC1 activity may be particularly critical for early growth phases of the thymus in ontogeny and during regeneration, when anabolic metabolism is required for generation of macromolecules for cell growth and division. mTORC2 activity may be more important during maintenance phases in TEC, when catabolic metabolic process such as autophagy are important for TEC function. As discussed below, extensive crosstalk between mTOR signaling, ROS, and autophagy has been described in diverse model systems, and this integration optimizes cellular responses.

## Redox Regulation of TEC Function

Reactive oxygen species (ROS) are generated as byproducts of cellular respiration (28), and may therefore be regulated by mTOR-mediated increases in metabolism (6, 29–31). ROS can also be generated by oxidative enzymes, detoxified by antioxidant enzymes, and when present at moderate levels, function as critical signaling molecules (32), including as important regulators of T cell receptor signaling (3). ROS are critical modulators of stem cell activity, including in intestinal epithelium (33) and bone marrow (34), when within moderate concentration ranges (6). At high levels, ROS can cause oxidative damage to cellular proteins, lipids, DNA, and other macromolecules (35), and oxidative damage has long been considered to be a primary cause of aging (36).

Several lines of evidence point to an unusual redox environment within thymic stromal cells. First, thymic stromal cells, notably cTEC, are continuously exposed to developing T cells undergoing especially high rates of cell division (37, 38). As a result, the stromal cells, unlike lymphoid cells which quickly exit the cell cycle and emigrate (39), will persist in a state of exposure to the cell-permeable products of high metabolic rates and cell division such as ROS (2), including H_2_O_2_, and may therefore experience particularly high ROS levels. Indeed, a similar scenario has been demonstrated in the bone marrow, where Cx43-depenent channels facilitate transfer of ROS from proliferating hematopoietic stem cells to adjacent bone marrow stromal cells, a function critical for hematopoietic regeneration (40). In addition, studies have shown that thymic stromal cells, especially those in the cortex, express conspicuously low levels of the H_2_O_2_-quenching enzyme, catalase (41). As a result, TECs are especially vulnerable to oxidative DNA damage, which accumulates in TEC at significantly higher levels than that found in thymic lymphocytes in mice (41) and humans (42). This oxidative damage is a major contributor to age-associated thymic atrophy, which is delayed by dietary or genetic complementation of catalase activity (41).

Given the cellular damage incurred as a result of oxidative stress, as well as the impact of oxidative damage on thymus size, it is somewhat surprising that catalase expression is found at such low levels in thymic stromal cells, and suggests a selective advantage for a highly oxidative environment within this population. Positive regulators of catalase expression include FOXO transcription factors, which are inhibited by AKT signaling downstream of many growth factors, including those that promote mTOR activation, such as IGF (28, 43, 44). In this way, the same ligands that activate mTOR in TEC, may also inhibit expression of catalase and other antioxidant enzymes and promote ROS production. Conversely, the unfolded protein response (UPR), which can be initiated by ROS (45), can negatively regulate mTORC1 activity (17). In the sections below, we consider the interactions between high levels of ROS, mTOR activity, and autophagy in the regulation of critical TEC functions in the steady state thymus.

## Autophagy in TEC

Autophagy is the process through which cellular components are degraded and shuttled to the lysosome in order to produce new building blocks during times of nutrient deprivation (46). In the immune system, autophagy is also considered an important regulator of inflammation and antigen presentation (47). Autophagy in thymic stromal cells is essential for presentation of self-antigens, positive and negative selection, and induction of central tolerance (15). Transplantation of autophagy deficient (Atg5 KO) thymi into athymic hosts results in aberrant T cell selection and profound autoimmune disease due to loss of central tolerance in the defective transplanted thymic microenvironment (15), and this result has been corroborated by studies using varying models of autophagy deficiency in TEC (48).

As mentioned above, cTECs partially recover the age-associated loss of their processes and labyrinth morphology during regeneration (13). These results indicate that cTEC morphology regulates overall thymus size, and is also likely to affect the cell surface area available for antigen presentation necessary for proper T cell selection. The observation that thymus size is regulated by cTEC morphology suggests a novel mechanism by which autophagy may regulate thymus function, in addition to the known roles in generation of self-peptide and antigen presentation. Autophagy has an emerging role in establishing and maintaining cellular morphology in a number of systems including macrophages in flies and mice (49), HeLa cells (50), and mouse mammary tumor models (51), where autophagy has generally been shown to promote cell spreading by promoting extension of F-actin protrusions, and turnover of integrins and focal adhesions, respectively. Together, these observations suggest that autophagy may regulate cellular projections that are critical for cTEC function via generation of extensive niches which may regulate antigen presentation and thymus size.

In TEC, unlike most other cells, autophagy is active constitutively, rather than being starvation-induced (15, 46). This is somewhat predictable for functions such as antigen presentation or maintenance of cell morphology, which are continuously required. However, the mechanisms regulating this constitutive activation of autophagy have not been identified in TEC. In other biological systems, ROS are known to induce autophagy [(2) and reviewed in (52)], with established roles during physiological stress [i.e., oxidation/activation of Atg4 during starvation (53)] and in disease [i.e., cardiac ischemia (54)]. This suggests that the constitutively high levels of ROS established by low catalase expression in TEC, especially cTEC, may promote the high basal levels of autophagy critical for their function.

## Crosstalk Between mTOR, ROS, and Autophagy

**Figure 1:**
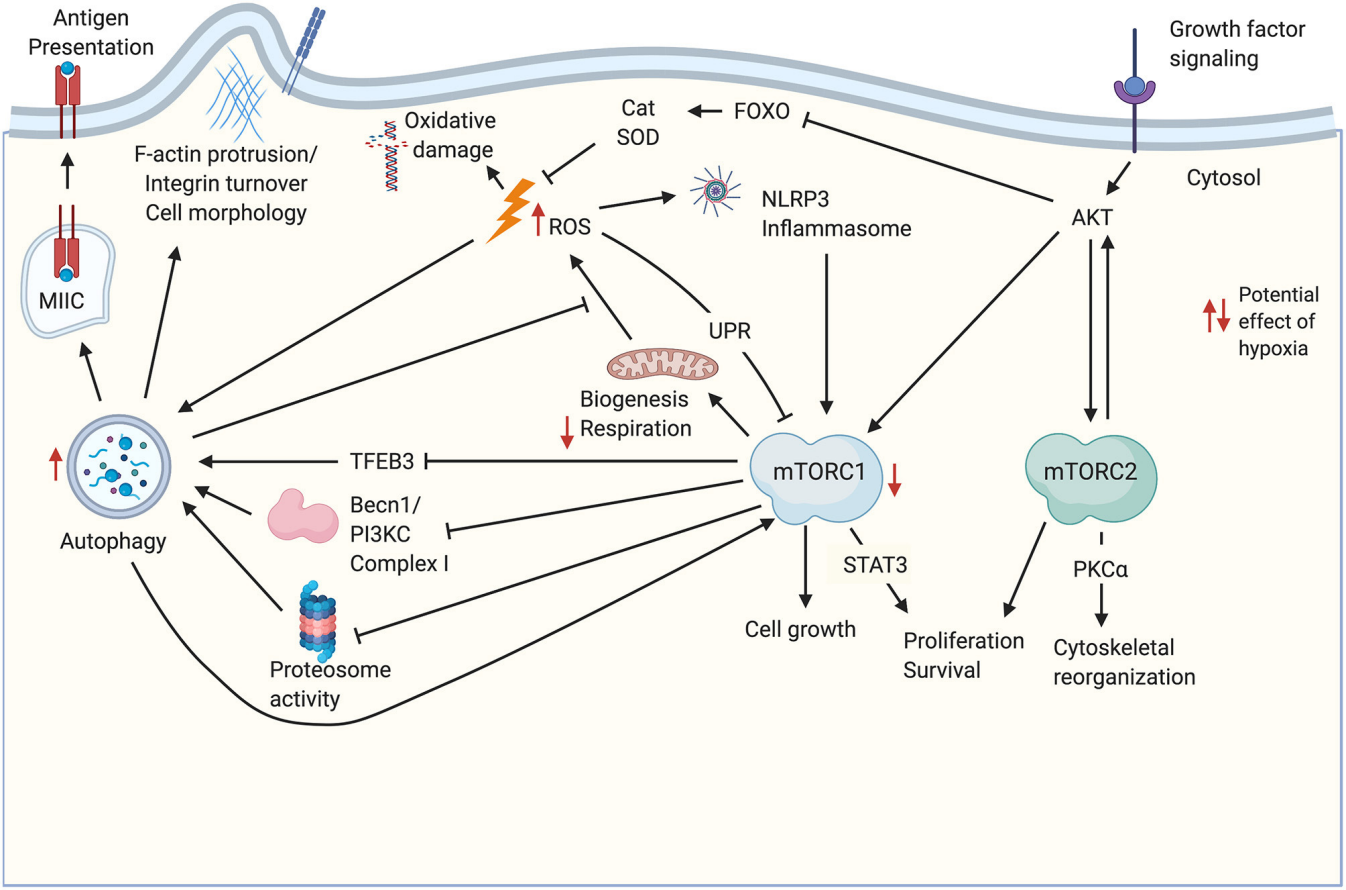
Potential cross-talk and co-regulation of mTOR signaling, ROS, and autophagy in TEC. Growth factor signaling induces AKT activation upstream of mTORC1/mTORC2 complexes. Both complexes promote cell proliferation and survival. mTORC1 signaling drives biogenesis including production of mitochondria required for oxidative phosphorylation. Increased metabolism leads to increased byproducts of metabolism including ROS, which can damage DNA and other macromolecules, including mitochondrial damage that exacerbates ROS production. ROS production is also promoted through the AKT-mediated inhibition of FOXO transcription factors that regulate antioxidant enzymes like Cat and SOD. ROS can feedback to inhibit mTORC1 activity via the UPR, and can activate the NLRP3 inflammasome. ROS promote autophagy, which also mitigates mitochondrial damage by increasing turnover of damaged organelles. Autophagy also promotes antigen processing and presentation in TEC, and may influence cell morphology, for instance, through turnover of integrins. Autophagy is mitigated by mTORC1 signaling via decreases in TFEB-mediated transcription of autophagy genes, as well as inhibition of proteosome activity and autophagosome assembly. mTORC2 phosphorylates several PKC family members, including PKCα, which regulate cell morphology and size through the actin cytoskeleton. Potential impacts of hypoxia are indicated by red arrows. Created with BioRender.com. Becn1-PI3KC Complex, Beclin 1- phosphatidylinositol 3-kinase complex; Cat, catalase; Foxo, class O of forkhead box transcription factors; MIIC, MHC class II-containing compartment; PKCα, Protein Kinase Cα; SOD, superoxide dismutase; STAT3, signal transducer and activator of transcription 3; ROS, reactive oxygen species; TFEB3, transcription factor EB; UPR, unfolded protein response.

Significant crosstalk occurs between the metabolic pathways described above, and the balance between them may be critical for thymic stromal cell function and maintenance. Some relevant potential interactions are highlighted in Figure 1. For instance, upon stimulation by an mTOR-stimulating ligand such as IGF1, growth factor receptor signaling initiates a kinase cascade that activates AKT (17). AKT activation leads to stimulation of the mTORC1 and mTORC2 complexes (17, 55), as well as inhibition of antioxidant activity via downregulation of FOXO-mediated transcription of enzymes like catalase and SOD (44), which would be expected to cause an increase in ROS. The consequences of increased ROS may include inhibition of mTORC1 through the UPR (17), stimulation of the NLRP3 inflammasome (56), and increased autophagy flux, for instance via activation of Atg4 (53). Increased ROS-mediated autophagy may in turn mitigate some ROS-induced cellular damage by increased turnover of damaged mitochondria (1), and may also promote self-antigen presentation required for T cell tolerance induction (15). This autophagic activity may in turn be antagonized by mTORC1 (12). In addition to the effects on antioxidant enzyme expression, activation of mTORC1 by AKT may also increase ROS by increasing metabolic flux as described above (6), however, mitochondrial biogenesis downstream of mTORC1 activation may balance this effect by producing healthy mitochondria to replace those that are damaged and may be a source of ROS (17). Notably, both ROS-induced oxidative damage (41) and NLRP3 inflammasome signaling (57) promote TEC damage during thymic atrophy.

The interaction of mTOR signaling, ROS, and autophagy should also be considered within the context of hypoxia. The thymus is hypoxic under physiological conditions (58, 59), and in fact hypoxia appears to promote thymocyte survival and development (58). This is consistent with studies showing that long-term repopulating hematopoietic stem cells are largely concentrated in hypoxic regions of the bone marrow (60). Little is known regarding the effect of hypoxia on TEC biology. Although stabilization of HIF1a represents a primary signaling pathway downstream of mTORC1 signaling under normoxic conditions (61), hypoxia also inhibits mTORC1 activity (62). This represents one way in which the downstream outcome and balance of mTORC1/mTORC2 signaling may be unique in hypoxic TEC, relative to other populations. In TEC, physiologically hypoxic conditions may generally inhibit mTORC1 signaling, while HIF1a stabilization, and therefore downstream signaling, is maintained by hypoxic conditions directly, independent of mTORC1. Another way the hypoxic steady state conditions in the thymus may affect the balance of mTORC signaling outcomes is by diminishing TCA cycle flux and downstream ETC flux [reviewed in (63)]. Increases in ROS mediated by low levels of O_2_ available as an electron acceptor (63) may influence ROS-mediated impacts on mTOR signaling. Hypoxia also promotes autophagy (64), which may allow for higher levels of autophagic flux in TEC under conditions favoring mTORC1 activity relative to cells under normoxic conditions. Potential impacts of hypoxia on mTOR activity, ROS, and autophagy are indicated in Figure 1.

On balance, the available data support a role for mTORC1 activation in promoting TEC proliferation during thymus growth (13, 22). Activation of mTORC2 would be expected to promote lipogenesis required for cell growth, and for cytoskeletal organization (16) that may be important for maintaining TEC morphology, consistent with published reports (13, 23). Given the physiological importance of mTOR signaling, ROS, and autophagy in TEC, as well as the highly interactive nature of these metabolic pathways, further studies will be required to unravel the mechanisms that regulate the balance of catabolic and anabolic processes in TEC. Such studies will be most informative when done in a physiological setting, *in situ*. The genetic and imaging tools required for assessing the morphology of individual TEC, autophagy flux, as well as selective ablation or promotion of the individual pathway components are emerging. For instance, by randomly activating expression of one of four potential fluorophores, Confetti mice (65) allow identification of individual cell morphology, including TEC morphology *in situ*. Beclin 1 knock-in mice (66) allow independent manipulation of autophagy flux, which can be visualized using RFP-GFP-LC3 fusion mouse models (67). mTORC1 and mTORC2 signaling pathways can be independently disrupted using floxed Raptor (68) and Rictor (69) alleles, respectively. ROS can be independently manipulated by overexpression or ablation of antioxidant genes such as Catalase (70). Studies exploiting models such as these may allow more comprehensive understanding of the basic biology of stromal cells in the steady state thymus, and facilitate the design of informed strategies for delaying and reversing age-associate thymus dysfunction.

## Author Contributions

MS, NJ, and AG researched, wrote, and edited the manuscript. All authors contributed to the article and approved the submitted version.

## Conflict of Interest

The authors declare that the research was conducted in the absence of any commercial or financial relationships that could be construed as a potential conflict of interest.
